# Breast Lesion Detection Using Weakly Dependent Customized Features and Machine Learning Models with Explainable Artificial Intelligence

**DOI:** 10.3390/jimaging11050135

**Published:** 2025-04-28

**Authors:** Simona Moldovanu, Dan Munteanu, Keka C. Biswas, Luminita Moraru

**Affiliations:** 1 The Modelling & Simulation Laboratory, Dunarea de Jos University of Galati, 47 Domneasca Street, 800008 Galati, Romania; simona.moldovanu@ugal.ro; 2 Department of Computer Science and Information Technology, Faculty of Automation, Computers, Electrical Engineering and Electronics, Dunarea de Jos University of Galati, 47 Domneasca Street, 800008 Galati, Romania; dan.munteanu@ugal.ro; 3 Department of Biological Sciences, University of Alabama at Huntsville, Huntsville, AL 35899, USA; kcb0015@uah.edu; 4 Department of Chemistry, Physics & Environment, Faculty of Sciences and Environment, Dunarea de Jos University of Galati, 47 Domneasca Street, 800008 Galati, Romania; 5 Department of Physics, School of Science and Technology, Sefako Makgatho Health Sciences University, Medunsa, Pretoria 0204, South Africa

**Keywords:** XAI, machine learning, LIME, SHAP, dependent features

## Abstract

This research proposes a novel strategy for accurate breast lesion classification that combines explainable artificial intelligence (XAI), machine learning (ML) classifiers, and customized weakly dependent features from ultrasound (BU) images. Two new weakly dependent feature classes are proposed to improve the diagnostic accuracy and diversify the training data. These are based on image intensity variations and the area of bounded partitions and provide complementary rather than overlapping information. ML classifiers such as Random Forest (RF), Extreme Gradient Boosting (XGB), Gradient Boosting Classifiers (GBC), and LASSO regression were trained with both customized feature classes. To validate the reliability of our study and the results obtained, we conducted a statistical analysis using the McNemar test. Later, an XAI model was combined with ML to tackle the influence of certain features, the constraints of feature selection, and the interpretability capabilities across various ML models. LIME (Local Interpretable Model-Agnostic Explanations) and SHAP (SHapley Additive exPlanations) models were used in the XAI process to enhance the transparency and interpretation in clinical decision-making. The results revealed common relevant features for the malignant class, consistently identified by all of the classifiers, and for the benign class. However, we observed variations in the feature importance rankings across the different classifiers. Furthermore, our study demonstrates that the correlation between dependent features does not impact explainability.

## 1. Introduction

Breast cancer (BC) is one of the most widely diagnosed types of cancer in women, with a high rate of occurrence and death. According to data reported for 2022, the highest occurrence rates were in China, with 357,161 cases, the United States (274,375 cases), and India (192,020 cases) [1,2]. An estimation for BC occurrence in 2024, as reported the American Cancer Society, suggests 310,720 cases, of which 56,500 are newly diagnosed cases, and 42,250 deaths. Globally, in 2022, approximately 2.3 million women were diagnosed with breast cancer, and 670,000 deaths were recorded [3].

Usually, breast cancer starts in the cells surrounding the ducts, glands, and mammary tissues that produce milk and in the ducts that transport milk [4]. The main risk factors for breast cancer include genetic predisposition, obesity, diet, tobacco and alcohol consumption, and high levels of estrogen postmenopause [5]. The risk of breast cancer increases with age. The most prevalent age group for breast cancer is women between the ages of 50 and 69 [6]. Breast cancer survival rates increase with early diagnosis, with a clinical rate of over 90% for early stages of detection. Magnetic resonance imaging, mammography, ultrasound, and positron emission tomography are the tools used to diagnose breast cancer. Usually, visual evaluation by a radiologist is used to evaluate tissue characteristics, detect abnormalities, discriminate lesions as a specific type, and make a diagnosis. This visual inspection is a sensitive method, and the diagnosis heavily relies on the radiologist’s skills.

ML algorithms extract meaningful features from the datasets and create a model to assess unfamiliar data. The most frequently addressed features include image texture with the first- and second-order features [7], Markov random field models [8], mathematic morphology [9], Hu image moment [10], fractals [11], geometric features [12,13], and radiomic features [14,15,16]. ML models handle these features as input data, and the classification or regression results serve to create a solution to the problem. Moreover, the integration of both genetic attribute information and temporal progression data holds significant potential for improving the predictive accuracy of machine learning algorithms [17]. However, ML models act as black box models, and their decision-making lacks accurate interpretation capabilities. ML models’ rules governing decision-making processes, such as how information is weighted, internal representation, and the final decision, are not accessible or understandable. Most of the time, it is critical to decipher and infer what an AI-assisted medical decision-making model’s data are deducing. XAI addresses this issue of transparency in AI systems. It evaluates the degree of explainability in AI models and has gained significance among researchers, particularly following the EU’s initiative to regulate AI [18]. Techniques like LIME and SHAP address the transparency and trust issues in ML models, reduce skepticism among healthcare practitioners, and facilitate the interpretability of the results. LIME [19] is a post hoc and model-agnostic explanation technique that estimates the decision boundary of a model as linear in the proximity of that instance, asking for an explanation. The LIME output consists of three parts: prediction probabilities, feature probabilities, and feature values. SHAP [20] is a post hoc and model-agnostic explainable model that works with feature-based datasets to pinpoint the pertinent variables and assess their influence on the prediction’s significance. Both models are “model-agnostic”, meaning they can be used with any ML model regardless of its architecture or underlying algorithms.

This research is driven by three specific motivations: (1) Some weakly dependent features derived from the image texture and lesion geometry are proposed. These provide complementary rather than overlapping information, improving the classification performance. One feature class is based on image intensity variations, and the second class uses the area of bounded partitions of the breast lesions. (2) Some sophisticated hard-to-interpret ML models, such as RF or gradient boosting algorithms, outperform the most interpretable linear or logistic regression models. In the first step, we assess the classification performance of a suite of ML classifiers when they are fed with the proposed weakly dependent features extracted from BU images. (3) Finally, in the third initiative, we thoroughly compare the explanations offered by LIME and SHAP regarding the impact and influence of the studied feature classes on the ML models. We address the issue of the different behaviors of complex ML models when they are exposed to feature combinations, i.e., to global or local behaviors, and how the prediction is distributed among the features.

The primary goal is to assess the ability of ML models, including Random Forest (RF), Extreme Gradient Boosting (XGB), Gradient Boosting Classifiers (GBC), and LASSO, to handle weakly dependent features and enhance the classification performance compared to conventional features. The following research objectives are the primary contributions:

- To propose new weakly dependent features, as follows: bounded histogram features (CHi) and grayscale density features (Ci). The dependency between the features is rarely analyzed, and this study investigates the impact of dependence within individual ML predictions and searches for explanatory reasons;
- To integrate XAI techniques like LIME and SHAP in ML models to understand the model’s predictions. XAI assists the classification process and enhances trust in distinguishing between benign and malignant breast lesions.
- To investigate ML and XAI models’ possible discrepancies in identifying the most important features and their explanations for decision-making contributions.

The rest of the paper is structured as follows. Section 2 reviews the state-of-the-art sound comparison with previously published research articles on the same subject. Section 3 highlights our approach for the weakly dependent features’ generation, hardware, software, and dataset; Section 4 provides an overview of the XAI models used to explain the ML systems employed for BC detection and classification, followed by the results and discussions in Section 5 and Section 6. Section 7 concludes the research.

Our method has been implemented in MATLAB R2021a and the feature datasets are available at the public repository https://github.com/simonamoldovanu/features_breast.

## 2. Related Work

In recent years, though numerous methods that enable the detection and diagnosis of breast cancer have been developed, the focus of most recent research has been on utilizing deep learning tools. However, a handful of studies have investigated the impact of dependence within individual ML predictions. Various ML-driven approaches using US images for precise and accurate BC diagnosis have been extensively developed [14,15,16,21,22,23,24]. Some baseline models, including SVM, RF, XGB, LightGBM, and voting classifiers, are trained using various databases and are optimized to enhance their performance. The feature importance (i.e., the individual contribution of each feature for a particular classifier) can be globally and locally explained. Improving AI explainability in clinical applications ensures that healthcare professionals can validate AI-generated insights, integrate them effectively into patient care, and comply with ethical and legal standards [25,26,27]. Table 1 presents the classifiers utilized in breast cancer diagnosis that provided the best values in terms of accuracy.

LIME [17] and SHAP [21,32] are two popular explainability tools that facilitate the explanation of ML classifiers’ predictions. Both models identify and quantify the features’ contribution to the model predictions but explain two different things. LIME uninterruptedly makes predictions explaining the difference between the prediction and a local average prediction, while SHAP explains the difference between the prediction and the global average prediction. Zhang et al. [32] examined various XAI medical diagnostic approaches, focusing on a case study demonstrating its application in accurate BC predictions and diagnoses. When the intrinsic XAI method (i.e., rule-based for the breast cancer classification task) was applied, the worst diagnosis performance (accuracy of 60.81%) was obtained. In contrast, post hoc XAI methods, such as SHAP and LIME, achieved the highest diagnostic performance, with an accuracy of 95.91% and a positive impact on predictions. Yagin et al. [33] investigated hybrid ML with SHAP models to predict metastatic BC. Based on the SHAP results, the most significant gene involved in predicting BC metastasis was identified. Lee et al. [34] predicted axillary lymph node metastasis and early BC with an accuracy of 81.05% using Mask R–CNN models. Then, LIME and the Simple Linear Iterative Clustering (SLIC) superpixel segmentation method were used to explain the prediction. The relevance of each superpixel image for a given classification was determined.

## 3. Experiment Setup and Weakly Dependent Feature Generation

### 3.1. Study Design

**Figure 1:**
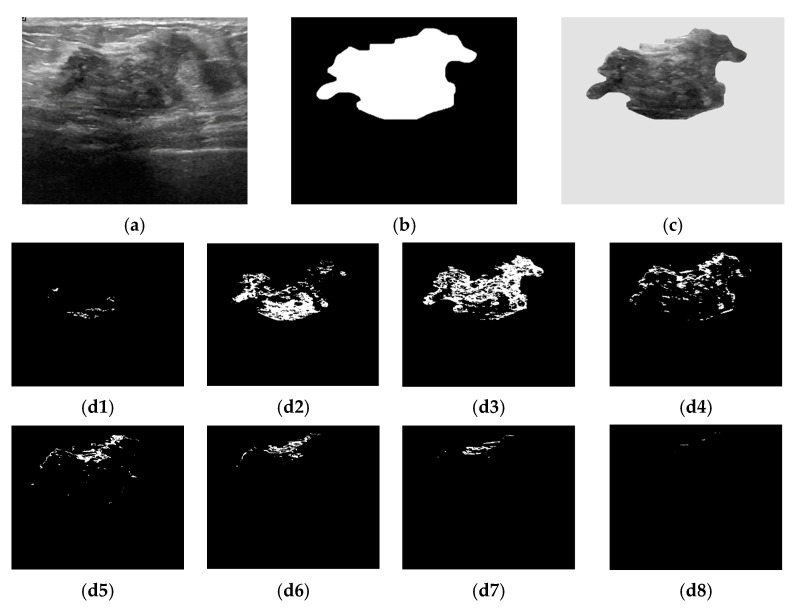
Bounded histogram features. (**a**) Raw breast US image from the BUSI dataset; (**b**) Ground truth of a breast lesion; (**c**) Region of interest. Selected pixels within bounded repartitions are shown according to their gray-levels distributions. (**d1**) [0, 31]; (**d2**) [32, 63]; (**d3**) [64, 95]; (**d4**) [96, 127]; (**d5**) [128, 159]; (**d6**) [160, 191]; (**d7**) [192, 223]; (**d8**) [224, 255].

We conducted our analysis on the data in the public BUSI dataset, created in 2018 and based on 600 female patients. It is publicly accessible at https://www.kaggle.com/datasets/sabahesaraki/breast-ultrasound-images-dataset (accessed on 1 March 2024) [35]. This dataset is divided into three classes: normal (266 images), benign (891 images), and malignant (421 images). In our study, only the last two categories were used along with their correct ground truth (clinical experts’ opinion of the segmented area of the lesion). A total of 438 benign and 211 malignant images and their associated ground truth images were selected. On average, the image size was 500 × 500 pixels. Each data point consisted of 16 features used as predictors. These features are shown in the following section. With the proposed architecture, the images from the BUSI dataset were split into “train” and “test” in a ratio of 75:25. A BUSI sample image is shown in Figure 1a,b. The specifications regarding the software, XAI models, and hardware used in our research experiment are presented in Table 2.

### 3.2. Weakly Dependent Features

#### 3.2.1. Bounded Histogram Features CHi

The histogram features were extracted from the breast lesion area (denoted I(m,n)) selected as the region of interest (ROI) (Figure 1c). The breast lesions differed in terms of their size, shape, and gray-levels distribution. As the image histogram correlates the pixel gray value and the number of occurrences, we proposed to split the histogram into a conjunction of bounded repartitions. Each repartition contains 2^5^ (=32) intensity levels [13]. The first feature class denoted as CHi is given by Equation (3). Figure 1(d1)–(d8) exemplifies the selected pixels based on whether they belong to a particular CHi feature.

(1) $$h\left(i\right)=\frac{1}{M\cdot N}\sum_{m=0}^{M-1}\sum_{n=0}^{N-1}\delta \left(i-I(m,n)\right),i=0,1,2,3,\ldots ,L-1$$

(2) $$\delta \left(i,j\right)=\left\{\begin{array}{ccc} 1 & if & x=y\\ 0 & if & x\neq y \end{array}\right.$$

(3) $$CH1=\sum_{i=0}^{31}h\left(i\right),\;CH2=\sum_{i=32}^{63}h\left(i\right),\;CH3=\sum_{i=64}^{95}h\left(i\right),\;CH4=\sum_{i=96}^{127}h\left(i\right)\;CH5=\sum_{i=128}^{159}h(i),\;CH6=\sum_{i=160}^{191}h(i),\;CH7=\sum_{i=192}^{223}h(i),\;CH8=\sum_{i=124}^{255}h(i)$$

#### 3.2.2. Grayscale Density Features Ci

In the breast cancer detection and diagnosis process, the size of the lesion is an important factor. To address the variation in the quality of the input images, the bounded repartitions CHi, i = 1, 2, …, 8, were grouped into coherent features by the ROIs’ geometric characteristics integration. Thus, the areas occupied by the pixels in each region (Chi) were determined, along with the area of the ground truth (GT) images (Figure 1b). The second dependent feature class, Ci, was determined as the ratio between the area of the bounded region (Chi) and the area of GT:

(4) $$Ci=\frac{area(CHi)}{{area}_{GT}},i=1,2,\ldots ,8.$$

#### 3.2.3. Algorithm for Extracting Bounded Histogram and Grayscale Density Features

To extract the CHi and Ci features, an in-house image processing algorithm was implemented. In the pseudocode (Algorithm 1), the following procedures are mentioned:

(i) The input consists of raw breast ultrasound (US) images (denoted as A) and the corresponding binary masks for each lesion (denoted as B), serving as the ground truth images. The output consists of two feature classes, one computed based on the image histograms and the other from the segmented ROIs (B superimposed over A).
(ii) To determine the CHi and Ci feature values, the sum of the intensity values of all pixels is computed as follows: sum(sum(B (:))) and sum(sum(ROI (:)).
(iii) The area of each bounded repartition histogram and the full ROI histogram is divided, allowing the computation of the eight CHi features using sum(H(:)).

**Algorithm 1.** Computation of CHi features**Input:** A<-grey level US images B<-ground truth images binary images ROI<-overleap(A,B) S = sum(sum(B (:))) H = histogram(ROI) **for** i: = 1 to 8 **do** p←0 j←31 **if** ROI >= p AND ROI <= j **then** Ci = sum(sum(ROI(:)))/S **end if**
**if** H >= p AND H <= j **then** Chi = sum(H(:)) **end if** p ← p + 32 j ← j + 32 **end for** **Output**: Eight Ci features Eight CHi features

## 4. AI Tools

### 4.1. ML Algorithms and Weakly Dependent Features—Importance

In ML practice, the features are rarely statistically independent. The dependent features influence the predictions and the feature contribution for individual predictions. Using the proposed weakly dependent features, we adopted RF, XGB, GBC, and LASSO classifiers for identifying the most significant feature [36]. The importance of feature selection and the robustness of an ML classifier are closely interrelated. Feature importance is determined based on the model error variations during the feature permutation process. A feature is important when its values permutation increases the model’s error values. A feature is irrelevant when the permutation process does not affect the model’s error. In this case, the feature was disregarded for prediction. Only a few features in the dataset were useful for model generation. The redundant variables impacted the model’s generalization capacity and impeded the overall accuracy of a classifier.

### 4.2. The Interpretation XAI Framework

To enhance interpretability in ML and facilitate new attributes for weakly dependent features, we implemented a quantitative interpretation framework for ML models. Integrating ML models with XAI techniques facilitates the understanding of how these models make predictions and enhances their clinical utility for BC classification. LIME is a local approach, while SHAP is a global explainer [19,20,31]. Both are attribution-based explanation models as they find and quantify the most important features of model predictions. The independence of the models’ explainability is known as model agnostics. In this study, the XAI models handled 649 data points from the BC dataset and eight features from each CHi and Ci category. They were labeled as malignant (0) or benign (1). The XAI techniques quantitatively evaluated the contributions of the most important selected CHi and Ci features, assessing their respective significance in the classifier’s outcomes.

#### 4.2.1. Local Interpretable Model-Agnostic Explanations (LIME)

LIME estimates the decision boundary complex models as linear in the proximity of that instance, asking for an explanation. Since LIME is a model-agnostic technique, it can be used with any classifier, whether it is linear or non-linear. To provide a trustworthy and explanatory interpretation, it learns locally weighted linear models using the neighborhood data of a particular observation [37]. LIME focuses on training RF, GBC, and XGB models using their specific hyperparameters and perturbing the selected CHi and Ci, i = 1, 2, …, 8 features to explain individual predictions. Furthermore, LIME gets a new black box prediction for these new/perturbed features and explains the most intriguing examples for both category features by interpreting the local model.

#### 4.2.2. SHapley Additive exPlanations (SHAP)

SHAP is an XAI model-neutral post hoc technique that works with any ML model. SHARP uses “Shapley values of the feature”, which represent the contribution of each feature to the prediction. Simply put, SHAP can describe the impact of features for both specific instances and all instances. It focuses on feature-based datasets to identify suitable features and estimate their influence on the significance of the prediction. SHAP relies on the hypothesis that each possible combination of features affects the overall prediction made by the model. Combinations of features and subsets of features are considered to account for all scenarios in the model to compute the feature influence scores [38]. Thus, a set of *n* features has 2*^n^* possible feature combinations. One feature is explained and the rest of the 2*^n^* − 1 combinations are evaluated.

## 5. Results

### 5.1. Classification Results

This paper investigates how the weakly dependent features influence the prediction and assesses and clarifies the feature contribution for individual predictions in the presence of feature dependence. It explains the model classification for more reliable and trustworthy results. Two dependent feature classes are proposed to increase the training data diversity and enhance the diagnostic accuracy. As the redundant variables impact a model’s generalization capacity and impede the overall accuracy of a classifier, a feature importance selection process was employed. Four algorithms and their parameters, namely RF (n_estimators = 200, criterion = ‘gini’, random_state = 0), XGB (random_state = 2, criterion = ‘friedman_mse’, learning_rate = 0.02, max_depth = 3), GBC (n_estimators = 100, learning_rate = 1.0, max_depth = 1, random_state = 0), and LASSO (penalty = ‘l1’, solver = ‘saga’, class_weight = ‘balanced’, max_iter = 10,000, all features were standardized with zero mean and unit variance, class weights were balanced to counteract any label imbalance with parameter class_weight = ‘balanced’ and up to 10,000 iterations with parameter max_iter = 10,000 were allowed, hyperparameter tuning was performed over the inverse regularization strength for C in {0.001, 0.01, 0.1, 1, 10, 100, 1000} and using 5-fold stratified CV for optimizing the ROC-AUC, the best-performing C was selected and then evaluated on the test set), were used to determine each feature’s importance. The classification of malignant and benign breast lesions in BU images was explained using LIME and SHAP.

To estimate the expected error rates of the proposed ML models, 5-fold cross-validation was used. Cross-validation assesses the error rate of a model by withholding a portion of the training data during model calibration and evaluating the performance on the excluded data. To ensure a more accurate performance evaluation, the benign and malignant groups should be evaluated separately, enabling a direct comparison of the sensitivities between the two classes [39]. The Chi-squared test shows that if the observed and expected values differ significantly, i.e., the Chi-squared value $\chi^{2}$ is large, a strong association between the variables exists. Table 3 reports the accuracy values and confusion matrices provided by each classifier and for the proposed CHi and Ci features. The classification results consistently yielded the most favorable outcomes. The RF model exhibited the superior performance in terms of accuracy, F1 score, area under the curve (AUC), and the results of the χ^2^ test.

For the proposed weakly dependent features, the RF classifier demonstrated a significant difference between the observed and expected values. The LASSO classifier performed poorly on both the CHi and Ci feature classes by omitting potentially important features from further analysis. Moreover, the LASSO classifier proved to be unstable during feature selection. In contrast, the RF and GBC algorithms demonstrated more robust feature selection capabilities. The χ^2^ test results collectively suggested a weak association between the variables. These findings highlight the necessity of exploring alternative methods for more effective feature identification and utilization.

**Figure 2:**
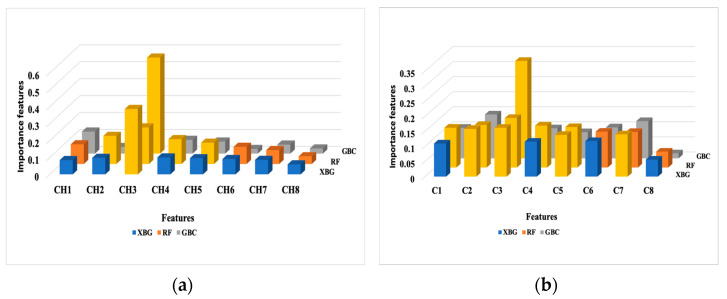
The overall feature importance in the prediction results over the test dataset. (**a**) Bounded histogram features (Chi); (**b**) Grayscale density features (Ci). The most important features are marked in yellow.

The overall feature importance estimated by the classifiers is displayed in Figure 2. A threshold of 0.125 was imposed as the average of the determined weight values for all considered features. The following results were returned by the classifiers: GBC (CH3), RF (CH2, CH3, CH4, CH5), and XGB (CH3) for bounded histogram features (Chi). In the case of grayscale density features (Ci), the selection process indicated GBC (C3), RF (C1, C2, C3, C4, C5), and XGB (C2, C3).

### 5.2. XAI Interpretability Results

The results of LIME integrated with ML models are presented in Table 4. For the CHi feature class, the XGB classifier predicted a malignant classification with only 13% probability while assigning 87% probability to the benign class. Similarly, the GBC classifier confidently classified samples as benign with a 96% probability, indicating that the CHi features provide a reliable basis for identifying benign breast conditions. In contrast, when using the Ci feature class, both the XGB and GBC classifiers showed increased uncertainty, predicting malignant cases with probabilities of 48% and 40%, respectively, and benign cases with 52% and 60%. The Random Forest (RF) classifier demonstrated moderate confidence with the CHi features, predicting 23% for malignant and 77% for benign cases. When using the Ci feature class, RF assigned 24% probability to malignant and 76% to benign. Notably, for the weakly dependent Ci feature class, all models were unable to provide high-confidence predictions for either benign or malignant conditions.

**Figure 3:**
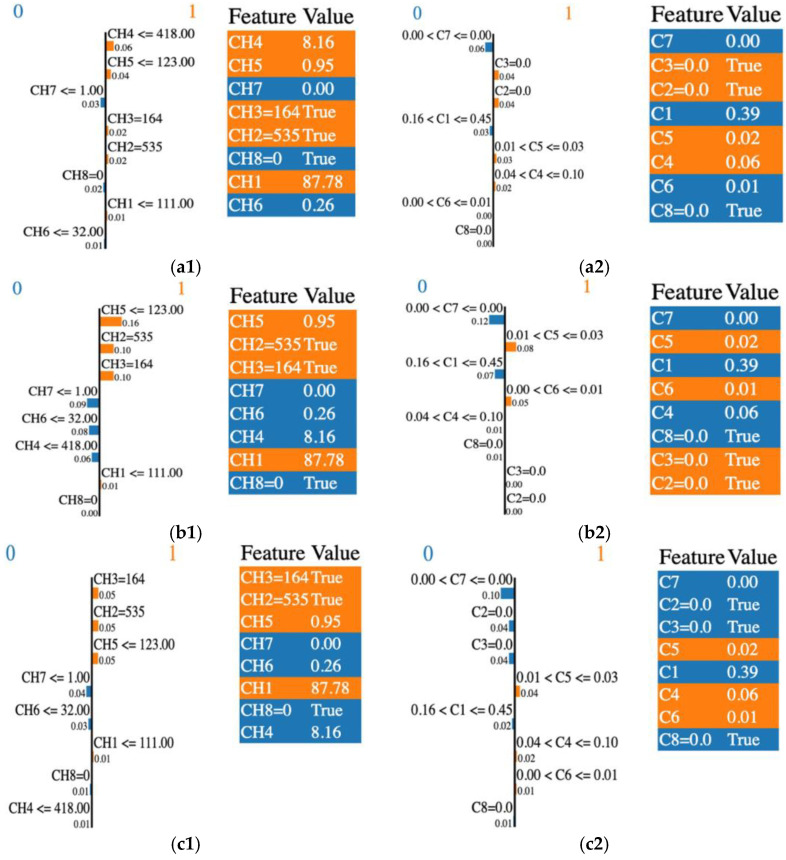
LIME output: the importance of individual features in the classification process by their relevance and score, and the features’ selection across various classifiers. (**a1**) RF and CHi; (**a2**) RF and Ci; (**b1**) GBC and CHi; (**b2**) GBC and Ci; (**c1**) XGB and CHi; (**c2**) XGB and Ci. “0” or blue is associated with the malignant class and “1” or orange is for the benign class.

LIME also explains the features’ contributions to a specific prediction. The LIME–integrated RF, XGB, and GBC outputs are shown in Figure 3. The feature probability graph displays how a feature affects a particular prediction. The number specified above the horizontal bar refers to the input variables’ index (CHi or Ci, i = 1,…, 8). The corresponding bar length is proportional to the contribution factor of the input-mentioned feature.

To illustrate how a sample is classified as malignant or benign, we analyzed the case of the Random Forest (RF) classifier. In Figure 3(a1), the RF algorithm identified CH4, CH5, and CH3 as the most influential features in predicting the benign condition (coded as 1), with contribution factors of 0.06, 0.04, and 0.02, respectively. Conversely, features such as CH7 and CH8 contributed to predicting the malignant condition (coded as 0), with contribution factors of 0.03 and 0.02, respectively; however, their influence remained minimal. Additionally, LIME highlighted the contributions of C3, C2, C5, and C4 in predicting the benign condition, as shown in Figure 3(a2). This finding aligns with the prediction probabilities presented in Table 4.

The most important contributing features that drive the prediction of malignant and benign classes are listed in Table 5.

To summarize, the common relevant features for the malignant class detected by all classifiers were CH7, CH6, and C1, C7, and C8. Similarly, the benign class was represented by CH1, CH3, CH5, and C5. We noted some differences in the importance rankings across the classifiers.

To provide insight into the LIME results, we recalled the RF case. CH2, CH3, CH4, and CH5 were selected as the most important features overall according to the feature importance in the RF classification (Figure 2). Among these, only the CH4 and CH7 features were identified with higher weights with LIME. Some features may have a lot of impact on individual predictions but may be split across the tree and thus be assigned low feature importance. In this case, we overcame the understanding of the RF model’s “typical” behavior by explaining the real contribution of some of the features to the prediction.

For the GBC and XGB classifiers, each boosting model provided access to their internal statistics as a form of feature weights. CH3 was selected as the most important feature by both models, while XGB selected C2, C3, and C5. Only C3 was selected by GBC, according to the feature importance presented in Figure 2. LIME’s top features shared with the top feature weights were different. Only CH3 and C3 were common features identified by these classifiers and LIME. The rest of the predictions were different. A good explanation for this behavior is the fact that LIME aims to explain single variables and their contribution to the final result, and is not a global model. Also, the LIME model contributes bias, especially in the feature space, where a high variance exists.

**Figure 4:**
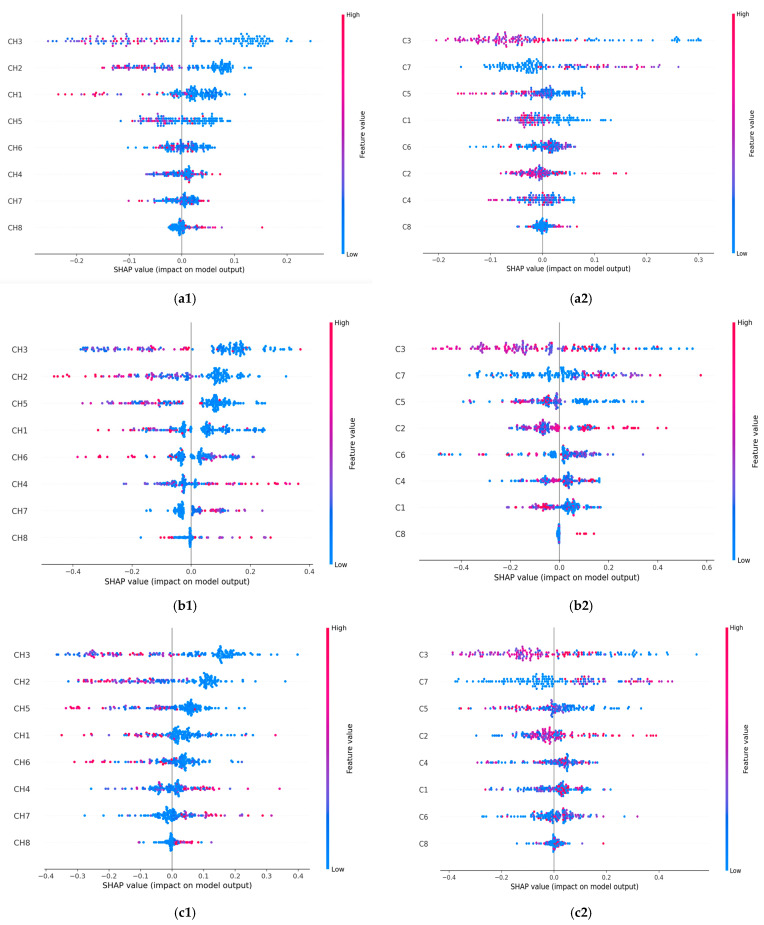
SHAP-integrated ML classifiers’ summary plot on the test data for the malignant and benign output classes. (**a1**) RF and CHi; (**a2**) RF and Ci; (**b1**) GBC and CHi; (**b2**) GBC and Ci; (**c1**) XGB and CHi; (**c2**) XGB and Ci. The horizontal axis plots an SV for a specific feature and data point. The vertical axis ranks the features based on their importance. The values of the features are represented with the following code: lower values are shown in blue, and higher values are shown in red. Points that overlap are shown vertically.

SHAP usually computes many Shapley values (SVs) and has fast implementation for tree-based models. SVs are calculated for each data point, corresponding to every input feature. The mean of the absolute SVs computed for each feature allows the features’ importance to be assessed. Figure 4 displays the plots of the SVs across the test dataset and for each classifier. The SVs are plotted for the CHi features (left column) and Ci features (right column) used as data points for the test set by each classifier. Higher SVs indicate a higher probability of a prediction. The lower values of the features reveal a malignant prediction while the higher values suggest a benign prediction.

Overall, the most important contributing features in the benign prediction were CH3 and CH2, along with C3. When the CH3 (or CH2) predictor had a high value of −0.2 (or −0.1), it positively affected the classifiers’ prediction of the benign breast condition and vice versa. In predicting malignant conditions, the CH8 and C8 predictors consistently impacted breast cancer identification. The prediction was positively affected by the higher values of 0.1 for CH8 and 0.02 for C8. Also, CH8, C3, CH2, C7, and CH1 had the highest number of contributing values. The most important contributing features driving the prediction of malignant and benign classes are listed in Table 6.

The SHAP values remained consistent when assessing the influence of a feature on the model’s output, as seen with the CH3 and CH8 features in Figure 2 and Figure 4. All classifiers identified CH3 as the most important feature in the model’s predictions (Figure 2). In the SHAP explainability process, CH3 consistently exhibited higher SVs when predicting the benign state, ensuring reliability. In contrast, while the classifiers rated CH8 as the least important feature, SHAP designated it as the most influential contributor to malignancy prediction. An explanation for this finding could be attributed to the irregular spiky margins of a typical malignant lesion, which also contains microcalcifications that usually appear lighter gray [40]. Proliferative breast disease is a common group of gray zone lesions that could include fibroadenoma transformation in ductal or lobular carcinomas, malignant phyllodes tumors, or simultaneous fibroadenoma and carcinoma appearing in the same breast. This finding provides insights into the importance of prioritizing more influential features. We also noticed that all classifiers selected C3 as the most important feature (Figure 2). In the SHAP explainability process, C3 had higher SVs in predicting the benign state; thus, consistency was assured (Figure 4).

Corroborating the information reported in Table 5 and Table 6, certain commonalities existed between the features recommended by the LIME and SHAP algorithms for breast conditions, as follows:

i. RF classifier—CH8 and C8 were the most important contributing features in the prediction of malignancy; CH3, CH1, C3, and C5 were the most important contributing features in the prediction of benign conditions;
ii. GBC classifier—CH7, CH8, CH4, C8, C1, and C4 were the most important contributing features in the prediction of malignancy; for benign conditions, they were CH3, CH2, CH5, C3, and C5;
iii. XGB classifier—CH7, CH8, CH4, C8, and C1 were the most contributing features in the prediction of malignancy; for benign conditions, they were CH3, CH2, CH5, and C6.

## 6. Discussion

This study revealed discrepancies between the ML and XAI models in identifying the most important features when weakly dependent features for breast lesion classification were utilized. It explains and analyzes their decision-making processes. These inconsistencies highlight the need for further investigation to understand their underlying causes. It is important to note that feature contribution values do not represent the true importance of a feature to the target outcome. Instead, they reflect what the model has learned during training based on the specific dataset and training process. As expected, the XAI techniques yielded different results, as their explanations are influenced by various factors, including classifier-specific randomization and tunable parameters. However, a good level of cohesion and consistency in the explanations was observed.

Based on the above analysis and the data in Figure 2, CH3 and C3 were consistently identified by trained classifiers as the most important features for predicting benign cases. LIME and SHAP confirmed both features as having a higher potential in the benign class prediction. This is likely due to the [64, 95] pixel range that contributes the most to classifying lesions as benign. C3 confirms this finding as a dependent feature, which calculates the area of the same lesion region. In the case of malignancy, both the LIME and SHAP explanations highlighted the CH7, CH8, C7, and C8 features as relevant for prediction. These lighter gray pixel ranges are related to the most malignant features in breast ultrasound images. We also observed that the correlation between the dependent features did not affect the explainability. On the contrary, it helped explain the XAI tools’ predictions by revealing a consistent trend in their outcomes.

The causes underlying the existing inconsistencies are the main challenge of XAI, as they could affect its reliability and applicability. The present study proposed a holistic approach to address these challenges within the context of the algorithms under consideration. The sources of the discrepancies are multiple, and we can also mention the complexity of the algorithms, the sensitivity of the feature importance related to the explanation approach of LIME or SHAP, and the complexity and non-linear interaction between the features that affect the interpretation of their behavior [41]. Also, we need to consider that LIME and the SVs explain different concepts through different approaches.

Regarding the classification performance, the RF algorithm provided the highest accuracy of 100% for both the CHi and Ci feature categories. Also, the RF algorithm was the best-performing model for breast cancer detection, while XGB showed lower performance (Table 3). This is possibly because RF combines multiple decision trees and can capture non-linear relationships within the data. RF is also robust to overfitting. XGB is sensitive to outliers as it is forced to fix the errors from the previous learners and is computationally expensive. Table 7 provides a short comparison with other results reported in the scientific literature.

By combining classification performance and explanation, we have proven that the performance of algorithms is required but not enough alone. That means the systems’ acceptance and trustworthiness increase when a satisfactory explanation complements their performance.

This study used a small dataset of breast ultrasonography images (BUSI), which may not be fully representative of the population and may be one of its limitations. The proposed approach will be extended to different datasets. Also, we limited our classification to malignant or benign without a detailed investigation of many types of lesions for both classes. This approach may alleviate the main disadvantage of the SHAP method, namely the computational cost. The necessity to balance explanation accuracy with simplicity when providing predictive insights defines another limitation.

In future work, we will develop multiple feature models and various classification algorithms (Automated machine learning, AutoML, is one of the solutions) and explore alternate XAI evaluation models (Class Activation Map and Gradient-weighted Class Activation Map CAM, GRAD-CAM, Permutation Importance PIMP, etc.).

## 7. Conclusions

This study introduced two new classes of weakly dependent features as inputs for three selected ML classifiers, incorporating XAI concepts relevant to breast cancer. LIME and SHAP served as complementary tools for addressing transparency and trust issues in ML model predictions. However, their outputs differed in identifying the most predictive features. LIME’s top predictive features did not fully align with the most important features selected by the classifiers, with only CH3 and C3 being consistently identified across both methods. This variation arose because LIME generated explanations based on individual data points from the test set. In contrast, SHAP, through SVs, highlighted CH8 and C8 as the dominant features for predicting malignancy, while CH3 and C3 were key for benign classification. Regarding consistency, our study found that when introducing correlated features into the dataset, SHAP’s explanations aligned more closely with the most important features identified by the classifiers than LIME. Additionally, our study highlights that the correlation between the dependent features did not affect the explainability. On the contrary, it helped explain the XAI tools’ predictions by providing a consistent trend in their outcomes.

## Figures and Tables

**Table 1:** Summary of studies evaluating the performance of machine learning algorithms in breast cancer diagnosis—accuracy results.

Reference/Year	Models	Dataset	Accuracy
[14], 2023	SVM	BreakHis	97.75%
[15], 2023	Logistic regression + SVM	Wisconsin Breast Cancer Dataset	96.50%
[16], 2024	LightGBM	Wisconsin Breast Cancer Dataset	95.00%
[18], 2021	SVM	Wisconsin Breast Cancer Dataset	96.50%
[21], 2020	SVM	Wisconsin Breast Cancer Diagnostic	98.00%
[22], 2023	XGBoost	BreakHis	99.27%
[23], 2024	XGBoost	Wisconsin Breast Cancer Dataset	94.74%
[24], 2024	RF	GSE9893	93.60%
[27], 2023	XGBoost	Mendeley	81.00%
[28], 2024	RF + SVM	Wisconsin Breast Cancer Dataset	99.99%
[29], 2022	LightGBM	Ultrasound breast images dataset	91.00%
[30], 2023	RF	Wisconsin Breast Cancer Dataset	98.60%
[31], 2022	Deep neural network (DNN)	Wisconsin Breast Cancer Dataset	97.00%

**Table 2:** Experimental setup for the proposed system.

Hardware/Software	Specification	Value
Hardware	Model	Mac BookPro
	Chip	Apple M1 Pro
	Memory	16 GB
Software	PyCharm 2023.3.3	Integrated development environment
	Python 3.10	Scikit-learn 1.4.1
		Shap 0.46.0 & Lime 0.2.0.1
	MATLAB R2021a	Image Processing Toolbox

**Table 3:** Classification performance and confusion matrix results. The performance metrics are the mean results of 5-fold cross-validation.

Feature Class	Classifiers	Accuracy	F1 Score	AUC	$\chi^{2}$	Confusion Matrix
CHi	XGB	0.851	0.739	0.728	2.66	[[34 16] [8 104]]
	RF	0.969	0.950	0.965	0.20	[[48 2] [3 109]]
	GBC	0.919	0.863	0.872	1.92	[[41 9] [4 109]]
	LASSO	0.776	0.834	0.798	0.482	[[28 14] [15 73]]
Ci	XGB	0.882	0.822	0.816	4.26	[[44 14] [5 99]]
	RF	0.969	0.957	0.967	0.20	[[57 2] [3 100]]
	GBC	0.938	0.901	0.916	0.40	[[46 6] [4 106]]
	LASSO	0.761	0.818	0.812	0.429	[[29 13] [18 70]]

**Table 4:** Predicted probabilities from the LIME-integrated ML classifiers in the test dataset.

Features	Classifier	Malignant (0) (%)	Benign (1) (%)
CHi	XGB	13	87
	RF	23	77
	GBC	4	96
Ci 2	XGB	48	52
	RF	24	76
	GBC	40	60

**Table 5:** Ordered list of relevant features ranked by importance, as predicted by LIME for each classifier and class. The common features are highlighted in boldface.

Classifier	Figure 3	Relevant Features	
		Malignant (0) (%)	Benign (1) (%)
RF	(a1)	**CH7**, CH8, **CH6**	CH4, CH5, CH3, CH2, CH1
	(a2)	C7, C1, C6, C8	C3, C2, C5, C4
GBC	(b1)	**CH7**, **CH6**, CH4, CH8	**CH5**, CH2, **CH3**, **CH1**
	(b2)	**C7**, **C1**, C4, **C8**	**C5**, C6, C3, C2
XGB	(c1)	**CH7**, **CH6**, CH8, CH4	**CH3**, CH2, **CH5**, **CH1**
	(c2)	**C7**, C2, C3, **C1**, **C8**	**C5**, C4, C6

**Table 6:** Ordered list of relevant features ranked by importance, as predicted by the SHAP-integrated ML classifier. The common features are highlighted in boldface.

Classifier	Figure 4	Relevant Features	
		Malignant (Blue)	Benign (Red)
RF	(a1)	**CH8**, CH7, CH4	**CH3**, CH2, CH1
	(a2)	**C8**, C4, C2	**C3**, C7, C5
GBC	(b1)	**CH8**, CH7, CH4	**CH3**, CH2, CH5
	(b2)	**C8**, C1, C4	**C3**, C7, C5
XGB	(c1)	**CH8**, CH7, CH4	**CH3**, CH2, CH5
	(c2)	**C8**, C6, C1	**C3**, C6, C1

**Table 7:** Predicted probabilities from logistic regression classifiers on the test dataset.

Reference, Year	AI/Accuracy	XAI	Dataset
Sathyan et al. [31], 2022	DNN/97%	SHAP, LIME	Wisconsin Diagnostic Breast Cancer
Uddin et al. [42], 2023	LightGBM/99%	SHAP	Kaggle Breast Cancer Dataset
Suresh et al. [43], 2023	XGB/98.42%.	SHAP	Wisconsin Diagnostic Breast Cancer
Maheswari et al. [44], 2024	RF/95.9%	SHAP, LIME	Kaggle Breast Cancer Dataset
Munshi et al. [28], 2024	RF+SVM/99.99%	SHAP	Wisconsin Diagnostic Breast Cancer
Kaushik et al. [45], 2024	SVM/97.90%	SHAP, LIME	Breast Cancer Dataset
Wani et al. [46], 2024	Light Gradient Boosting Model/98.71%	SHAP	SEER Breast Cancer dataset
Proposed	RF/100%	SHAP, LIME	BUSI dataset

## Data Availability

The databases used are publicly available at the following links: BUSI image dataset link: https://www.kaggle.com/datasets/sabahesaraki/breast-ultrasound-images-dataset, accessed on 1 March 2024. The feature datasets are available at the public repository https://github.com/simonamoldovanu/features_breast.
